# Prediction of titratable acidity of sour cream by Fourier-transform infrared spectroscopy

**DOI:** 10.3168/jdsc.2026-1101

**Published:** 2026-08-24

**Authors:** Duy Trinh, David McIntosh, Elizabeth Eckelkamp, Jiajia Chen, Alejandro Molina-Moctezuma, Tong Wang

**Affiliations:** 1 Department of Food Science, University of Tennessee, Knoxville, TN 37996; 2 Department of Plant Sciences, University of Tennessee, Knoxville, TN 37996; 3 Department of Animal Science, University of Tennessee, Knoxville, TN 37996; 4 School of Natural Resources, University of Tennessee, Knoxville, TN 37996

## Abstract

Fourier-transform infrared spectroscopy has become an increasingly valuable analytical tool in the dairy industry because it is fast, chemical-free, and can capture complex chemical information. However, predictive models for many cultured and formulated dairy products remain underdeveloped. This work evaluated the feasibility of using Fourier-transform infrared spectroscopy combined with chemometric modeling to predict an important quality attribute: titratable acidity of sour cream. This attribute is critical for product quality, regulatory compliance, and process efficiency, yet current reference or chemistry methods are labor-intensive, destructive, and limited in throughput. Commercial sour cream products were sampled weekly over 9 wk to capture batch-to-batch variability. Titratable acidity was determined by standard NaOH titration, and spectra were collected with the FOSS MilkoScan FT3. Partial least squares regression model achieved training and internal validation R^2^ values of 0.601 and 0.581, respectively, demonstrating that the spectra contain meaningful information about acid concentration. External validation yielded an R^2^ of 0.463, indicating that the model captured the general relationship between spectra and measured values, but was limited by formulation differences and sample diversity. As proof of concept, these findings suggest that expanded datasets and formulation-specific modeling strategies are needed for robust industrial applications.

## Graphical Abstract

**Figure:**
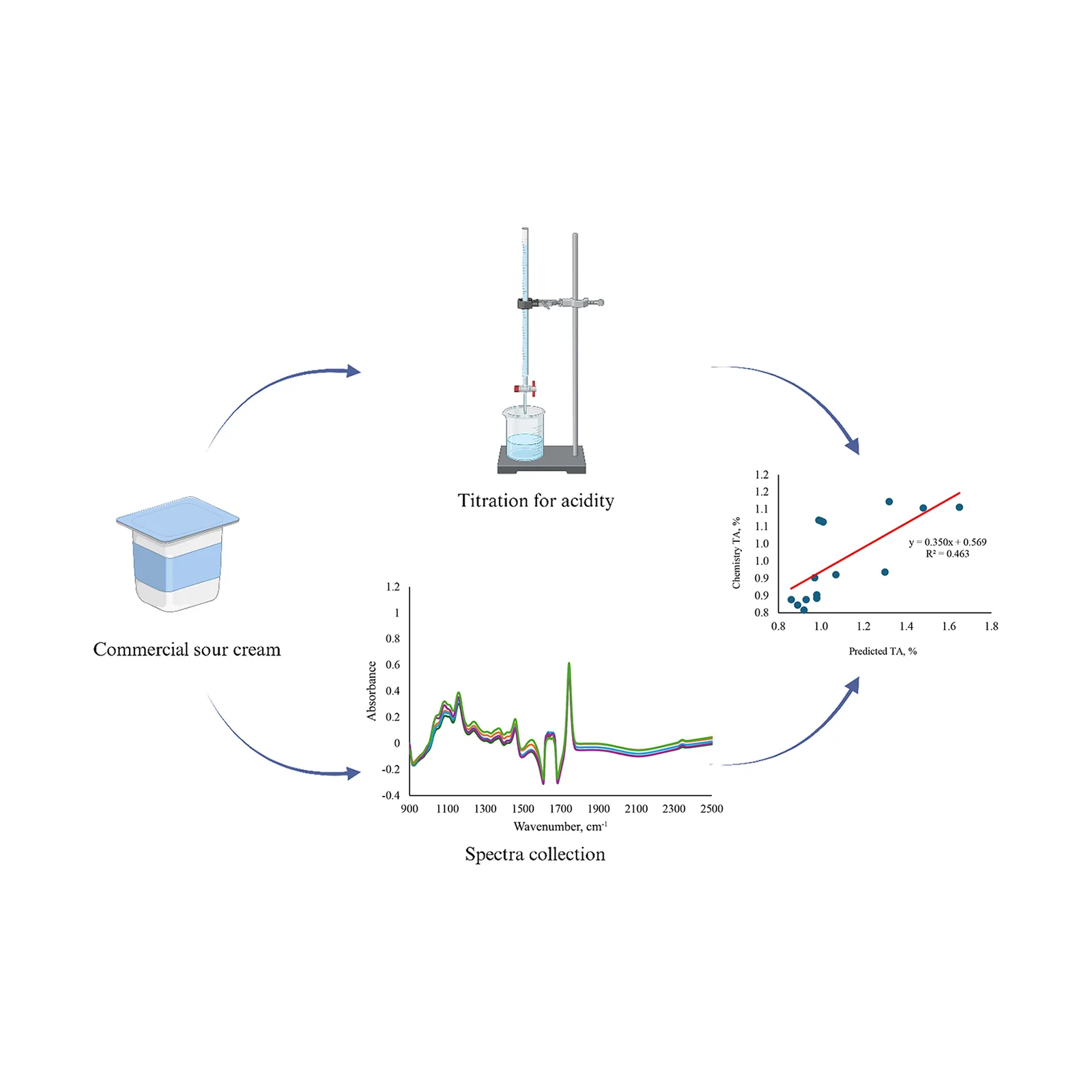

Titratable acidity (**TA**) is the total acid concentration in food. Commonly, food acids are organic acids (citric, lactic, tartaric, acetic, malic, and so on). The acid concentration affects food flavor due to its partial ionization and is determined by neutralizing the acid present in a known quantity (by weight or volume) of the food sample with a standard base. The endpoint for titration is typically either a target pH or the color change of a pH-sensitive dye, typically phenolphthalein. The volume of titrant used, along with the normality of the base and the volume (or weight) of the sample, is used to calculate the TA (i.e., the amount of titratable acid in a given amount of food expressed in terms of the predominant organic acid; Nielsen, 2017). This is a standard method (AOAC International, 2023), but it requires the use of a chemical reagent and is destructive and time-consuming. There is a need for a fast and accurate method of TA measurement to meet the industry's demand for quality control and research and development of large-scale production. Infrared (**IR**) spectroscopy prediction models can satisfy these requirements.

Infrared spectroscopy is a technology that analyzes the interaction of molecules with light in the infrared wavelength range (0.7–1,000 µm). These interactions include stretching, bending, or rocking of molecular bonds when exposed to IR electromagnetic radiation. The amount of chemical bonds in a given sample or product can be determined by the absorption signals in the spectrum, where stronger signals indicate a higher concentration (Pu et al., 2020). From the spectral profiles, it is possible to detect and quantify the chemical composition of samples through chemometrics. Chemometric techniques are used to analyze spectra and develop predictive calibration models from reference chemical data. Regular validation and calibration of these prediction models are required to ensure accuracy (Pu et al., 2021). Infrared spectroscopy is widely used in the food industry for quality control and analytical scanning because it is a rapid, accurate, and reagent-free method, especially when models are properly developed, compared with traditional analytical chemistry methods. The standard method for determining TA using NaOH titration (Calamari et al., 2016) was implemented for this study.

Sour cream is a high-acid food (pH <4.6). According to the Code of Federal Regulations, sour cream has TA of not less than 0.5%, which is a result of lactic acid bacteria fermentation. The TA of sour cream is calculated as lactic acid content [21 CFR 131.160 (a); https://www.ecfr.gov/current/title-21/chapter-I/subchapter-B/part-131/subpart-B/section-131.160]. The MilkoScan FT3 (FOSS, Hillerød, Denmark) has calibration models and up-to-date databases established for milk components (fat, protein, TS, SNF, lactose, glucose, galactose, density, urea, TA, free fatty acids, casein, lactic acid) for other dairy products, but specific models for sour cream's acidity are not available, and there is an industry-identified need to add such a model to its existing instrument and monitor acidity change during production.

In this study, sour cream was subjected to spectral scanning with Fourier-transform infrared spectroscopy (**FTIR**) and to TA determination by titration. Data from FTIR and titration were used to establish a quantitative calibration model. The specific objective of this work was to prove the concept before starting a much larger set of samples for industrial-scale modeling.

The following sour cream products were selected for model training: Daisy, Publix Regular, Kroger Original, Food Club, and Great Value. Each product was purchased once per week for up to 9 wk of sampling to ensure products were from different manufacturing batches. Each product was split into 3 subsamples. Subsamples were measured for TA and scanned for spectra on the same day of purchase. For external validation of models, another set of 5 products was purchased, measured for TA, and scanned for TA prediction with the new model to assess model performance.

Samples were thoroughly mixed with a spatula, then collected from different locations within the same container to ensure representative sampling. The TA was determined as described by Tesfay et al. (2015) with modifications. A 10-g sample of homogenized sour cream was collected and diluted with 10 mL of deionized water. Approximately 3 drops of phenolphthalein were added to the mixture. The mixture was mixed with a magnetic stirrer at 300 rpm for 5 min, then titrated with 0.1000 *N* Certified NaOH (Fisher, Waltham, MA) until a pink coloration appeared. A pH electrode (Mettler Toledo, Columbus, OH) probe (calibrated using standard solutions with pH 4.01, 7.00, and 10.01; Fisher, Waltham, MA) was also used to ensure consistency of titration with the determined end point of about pH 9.4. Each commercial product was sampled and titrated 3 times. Percent lactic acid was calculated using the following equation:

lacticacid(%wt/wtwtwt)=volumeofNaOH(mL)×0.1000N×90.08×100exactmassofsample(&sim;10g)×1,000.

Spectral data of sour creams were collected using the MilkoScan FT3 (FOSS, Hillerød, Denmark). An aliquot of 20 g from each subsample was taken and diluted with 20 mL of MSc Diluent (FOSS, Hillerød, Denmark, a proprietary liquid created to properly homogenize samples and lubricate the pumps of the instrument). Each subsample was measured once. Spectral collection and TA were performed on the same day to minimize product variation due to storage. Collected spectral data were stored in FOSS Manager Software (FOSS, Hillerød, Denmark) for further analysis.

The composition of sour creams was determined in 2 different ways (intended for studying the relationship between TA and composition): wet chemistry analysis using standard and official methods, and predictions from the existing FOSS model that shows high precision in predictions (R^2^ of 0.99 between the determined and predicted values). To assess the correlation of TA with protein, fat, lactose, TS, and SNF, data collected from chemical analyses and existing prediction models were used to establish multiple linear regression models. Models were validated by using 5-fold splits (4 for training and 1 for testing) and checked for linear model assumptions and predictor selection based on R^2^, Akaike information criterion (**AIC**), and variance inflation factor (**VIF**) values (data not shown). All statistical explorations were performed using R version 2024.12.0 build 467 (R Core Team, 2020). The following packages were used: car (Fox and Weisberg, 2018) and AICcmodavg (Mazerolle and Linden, 2023).

A quantitative calibration model for TA was generated using FOSS Calibrator Pro Software 3.9.2.10 (FOSS, Hillerød, Denmark). Briefly, the partial least squares (**PLS**) method was implemented to assess the correlation between TA from chemistry and spectral data, with 80% of the total data points (109) randomly selected for the training set and the remaining 20% (26 data points) for internal validation. Root mean square error of prediction (**RMSEP**) and R^2^ were reported for training, cross-validation, internal validation, and external validation sets of each model and were used to select models. The following information were collected: mean of the reference data, standard deviation of the reference data, method of developing calibration model, method of calibration evaluation, number of samples used for calibration and testing, mathematical data pre-treatment, number of factors, standard error of calibration, and standard error of cross-validation (Williams et al., 2017).

The products sampled for 9 weekly spectral scanning and titrations are shown in Table 1 and the average spectra of the 5 types of products are shown in Figure 1A. The TA data are the mean and CV over 9 wk of sampling. The high CV values indicate variations among batches from the same manufacturer. Such variation reflects normal batch-to-batch differences within the brand and type of commercial products and provides the diversity needed to develop calibration models. Incorporating samples that span the expected range of commercial variability is essential for improving model robustness and predictive performance (Fichman, 1999).

**Table 1:** Titratable acidity (TA) of commercial sour cream products used for modeling

Sample	Mean TA, % (n = 9)	CV, %
Publix Regular	1.11	7.80
Daisy	0.82	8.82
Kroger Original	0.88	10.43
Great Value Original	0.87	5.81
Food Club	1.00	9.47

**Figure 1:**
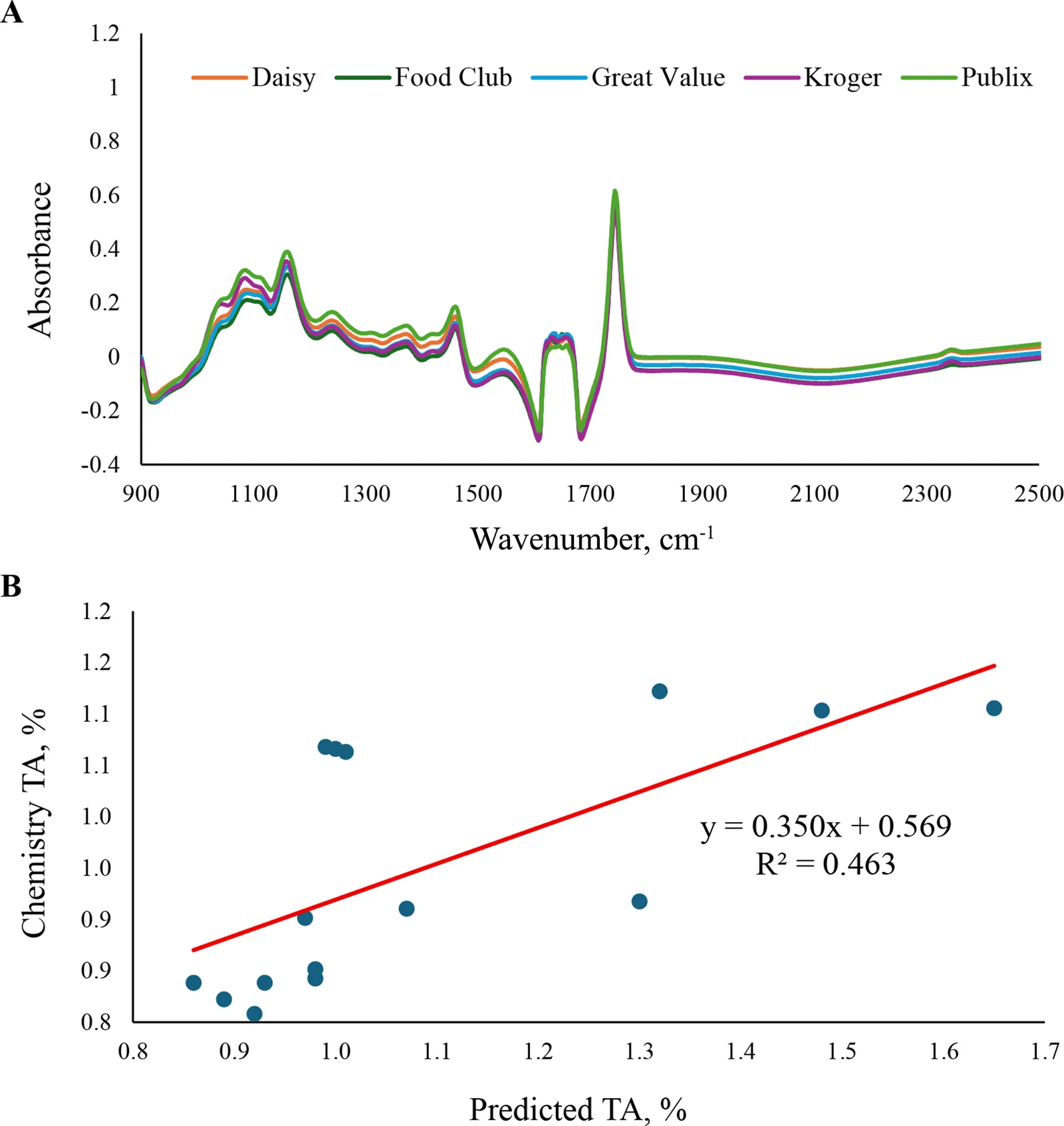
Spectra (averaged from 3 subsamplings and across 9 sampling times) from commercial sour cream used to build model (A) and chemistry TA versus predicted TA of sour cream samples used for external validation (B).

A PLS calibration model was developed to predict TA in sour cream using the MilkoScan FT3 mid-infrared spectra. The dataset consisted of 109 training samples and 26 internal validation samples, spanning a TA range of 0.74% to 1.22% in the training set and 0.75% to 1.22% in the validation set. The model used no scatter correction or derivatives, relying on raw absorbance features in 2 spectral regions based on the specific spectral peaks associated with TA: 1,052 to 1,142 cm^−1^ and 1,184 to 1,538 cm^−1^ (Figure 1A). According to the calibration report, the model employed 16 PLS factors, which is relatively high for a univariate prediction problem. High factor counts are common in the feasibility stage of dairy quality calibrations, especially when the analyte contributes only modestly to the overall spectral variation or when matrix effects dominate the spectra (Workman and Weyer, 2007; Ciurczak et al., 2021).

The absence of spectral pretreatment combined with a high factor count suggests that the model is capturing subtle spectral variation but may also be compensating for noise or collinearity. This is typical in feasibility stage calibrations, where the goal is to determine whether a predictive relationship exists before applying optimization steps such as pretreatment tuning or factor reduction.

The spectral regions selected by the calibration model are chemically relevant to the composition of fermented dairy products. The region between 1,052 and 1,142 cm^−1^ is primarily associated with C–O stretching vibrations of carbohydrates, including lactose and organic acids, whereas the 1,184 to 1,538 cm^−1^ region contains absorption bands attribute to C–O, C–N, and amide II vibrations associated with carbohydrates, proteins, and organic acids (Workman and Weyer, 2007). Although lactic acid is the primary contributor to TA, the measured TA also reflects the buffering capacity of proteins, phosphate salts, and other constituents. Consequently, the relationship between FTIR spectra and TA is a result from multiple chemically relevant components rather than a single analyte. This agrees with previous studies demonstrating that IR calibration models for dairy products rely on spectral information distributed across multiple absorption bands and one should explore covariance among chemically related constituents through multivariate calibration rather than individual diagnostic peaks (Pu et al., 2020). Therefore, the spectral regions selected by the PLS model provide a reasonable chemical basis for the observed predictive relationship between FTIR spectra and TA.

The calibration statistics indicate moderate predictive ability. The training set achieved an RMSEP of 0.082% and an R^2^ of 0.601, with a slope of 0.970 (Table 2). Based on general chemometric benchmarks, R^2^ values between 0.60 and 0.80 are typical for feasibility stage models where the analyte and spectral relationship is present but not strong (Williams and Norris, 1987). Cross-validation with the Venetian blinds method achieved RMSEP of 0.109% TA and R^2^ of 0.777, suggesting that the model captured meaningful spectral information but was limited by sample diversity and the absence of spectral pretreatment. The training bias was negligible (bias = −0.003), indicating no evidence of systematic over- or underprediction in the calibration set.

**Table 2:** Performance metrics of the sour cream model

Item	RMSEP,^1^ %	Bias	Slope	Intercept	R^2^
Training	0.082	−0.003	0.970	0.024	0.601
Cross-validation	0.109	0.009	0.942	0.060	0.777
Internal validation	0.086	0.010	0.823	0.174	0.581
External validation	0.048	0.139	0.350	0.569	0.462

1 RMSEP = root mean square error of prediction.

Although the calibration R^2^ is not high, the slope close to 1.0 suggests that the model captured the general linear trend between spectra and TA. The relatively low RMSEP compared with the TA range (∼0.5% variability) indicates that the model was able to resolve meaningful differences in TA, but with limited precision.

Internal validation from the 26 samples showed performance similar to cross-validation, with an RMSEP of 0.086% TA and R^2^ of 0.581, indicating moderate predictive capability. The bias achieved was negligible (0.010), confirming that systematic error remained low. These values fall within the range commonly reported for dairy FTIR calibrations of difficult-to-predict traits. For example, FTIR prediction of physiological or energetic traits in milk often yields R^2^ values between 0.50 and 0.85 (Rachah et al., 2020), whereas PLS models for milk fatty acids and cheese yield frequently fall between 0.30 and 0.75 (Ferragina et al., 2015). Even advanced machine learning models for milk traits often report R^2^ values of 0.20 to 0.80, depending on analyte strength (Mota et al., 2021). These comparisons indicate that the performance of the present TA model aligns with expectations for early FTIR calibrations in complex dairy systems.

An external validation set of 15 samples was chemically or analytically measured for TA and predicted with the newly generated model. Predicted TA was plotted against the reference chemistry TA and achieved an R^2^ value of 0.463 (Figure 1B). The model achieved an RMSEP of 0.048% TA, indicating that the average prediction error on new, unseen samples was relatively small. However, a positive bias of 0.139 showed that the model consistently overpredicted TA across the validation set, suggesting a systematic offset rather than purely random error. This bias could be due to variations in production and needs to be accounted for to ensure accurate prediction in industrial settings. Although R^2^ is low, the trend and clustering were observed. This is likely due to the low number of samples in the calibration set, resulting in limited representation and low diversity, so the new model could not predict TA accurately from different external samples. However, predictions showed that a potentially successful expansion of the calibration models is possible.

Sour cream also presents additional challenges, including fat globule scattering, stabilizer differences, and syneresis, all of which introduce spectral noise that can obscure acid-related absorbance features. This can be improved by collecting more spectral and reference chemistry data for the calibration set to cover larger variability when generating improved models. For a feasibility study, this level of performance demonstrates that the spectral regions contain relevant chemical information for TA prediction. Although FTIR has strong potential to predict TA in sour cream, further refinement is required before industrial-scale applications.

To further explore the statistical correlation between TA and other components (fat, protein, lactose, SNF, and lactic acid), multiple linear regression models were fitted to 2 compositional datasets (predicted and wet chemistry). The linear model was implemented by the equation below:

$$<!--ERROR-14INMATHTYPECONVERSION.PLEASECORRECTMANUALLY-->\$y_{i}=b_{0}+b_{1}x_{1i}+b_{2}x_{2i}+b_{3}x_{3i}+\ldots +b_{n}x_{ni}+{\;}_{i},\$$$

where *y_i_* is the response variable (TA); *i* is the sample number; *x*_1_, *x*_2_, and *x*_3_ are the explanatory variables (fat, protein, SNF, lactose, and lactic acid); *b*_0_ is the intercept coefficient; *b_i_* are the linear effect coefficients; and *ε_i_* is the error. Across all fitted models using FT3-generated data, the R^2^ values were very similar (approximately 0.69), which was expected given the relatively small sample size (135 observations). Variance inflation factors were used to assess multicollinearity. In the full model containing fat, protein, SNF, lactose, and lactic acid, VIF ranged from about 1.7 for fat to over 30 for lactose, indicating substantial collinearity among several predictors. These results agree with previous studies showing that dairy compositional traits, especially lactose, SNF, and protein, tend to be strongly correlated because they arise from shared biological and processing pathways (Ferragina et al., 2015; Mota et al., 2021).

A series of reduced models was evaluated to determine whether removing one or more predictors can improve model stability or provide a more efficient structure. Most reduced models produced R^2^ values within 0.002 to 0.01 of the full model. This demonstrates that the explanatory power of the model was robust. This pattern is consistent with previous work showing that TA in cultured dairy products is influenced by several compositional factors rather than a single dominant component (Calamari et al., 2016; Pu et al., 2020).

The AIC values were used to compare model fit while penalizing model complexity. The full model without lactose produced the lowest AIC, indicating the best balance between explanatory power and parsimony. However, one reduced model showed a protein VIF greater than 10, indicating a level of multicollinearity that raised concerns about the stability of the coefficient estimates. After excluding this model, the next-best model was the version that excluded both fat and lactose, which maintained a similar R^2^, had a lower AIC, and met multicollinearity requirements. These results suggest that protein was the most stable and informative predictor of TA when using the FT3-generated data. These findings are consistent with established literature showing that protein content is often associated with acid development and buffering capacity in fermented dairy systems (Workman and Weyer, 2007; Rachah et al., 2020). The strong collinearity observed among lactose, SNF, and lactic acid reflects the biochemical pathway of fermentation, in which lactose is converted to lactic acid and contributes to changes in solids content. Similar patterns have been reported in IR-based modeling of milk acidity, fermentation traits, and cheese yield, where interdependence among predictors complicates regression modeling (Ferragina et al., 2015; Mota et al., 2021).

Models based on the chemistry data were limited in interpretability due to aliasing among calculated variables and high variability introduced by frozen storage (for a few months) after the TA measurement and spectra collection and the uniformity problem of sample matrix. Hence only the predicted composition data were used for the TA-composition relationship study. This matrix uniformity change is consistent with previous reports showing that physical changes during storage can alter the distribution of fat globules, protein networks, and serum phases, which in turn affects chemical measurements (Pu et al., 2021). These limitations highlight the need for careful sample preparation and consistent analytical procedures when developing prediction models. Despite these limitations, the combined results indicate that TA of sour cream is influenced by several compositional factors (fat, protein, lactose, and other components), and that careful evaluation of predictor collinearity is essential when fitting regression models.

In summary, this study investigated the feasibility of using FTIR spectroscopy combined with chemometric modeling to predict TA of sour cream. The results demonstrate that FTIR contains meaningful chemical information related to this attribute, but also reveal the challenges of modeling complex, heterogeneous dairy matrices with limited calibration datasets. The models achieved moderate predictive performance, with calibration and validation statistics indicating that FTIR can detect spectral features associated with lactic acid content. Although the external validation set produced lower predictive accuracy, the observed linear trend confirmed that TA is spectrally encoded and can be modeled with expanded datasets. The study also highlights the influence of formulation differences and impact on spectral variability and the need for formulation-specific or more diverse calibration sets. This report shows the importance of continuing the work with more samples that have large degree of composition variations to ensure robustness of the prediction. It would be valuable to also study if gentle mixing and dilution of dairy products having thick and semisolid texture can be avoided to make in-line measurement possible. It is expected that this work will lead to more funding investment and collaborative efforts to ultimately achieve commercial application.
